# Reports on Hospitals of the United Kingdom

**Published:** 1914-02-21

**Authors:** Henry Burdett


### REPORTS ON
### Hospitals of the United Kingdom.

By SIR HENRY BURDETT, K.C.B., K.C.V.O.

SERIES III.

### THE ST. ALBANS AND MID-HERTS HOSPITAL.

This institution was commenced as a dispensary in 1843. A small hospital was added in 1870. In 1912 the hospital was enlarged, the changes made including additions to the women's and men's wards, which gave an extra two beds to each, and the provision, over the women's ward, of a new ward to contain ten beds, or fifteen cots, the reconstruction of the bathrooms with new bath, an isolation ward, and additional nurses' accommodation. A handsome, spacious, and fully-roofed verandah, being, in fact, an open-air ward, was added along the south side of the hospital. The furniture and equipment is smart, and the whole appearance of the wards, their proportions, lighting, and hygienic conditions are excellent. The sisters and nurses have comfortable quarters, and each nurse has a separate bedroom.

The hospital, as reconstructed, contains forty beds. The dispensary is situated in the main buildings on the right side as one enters the hospital grounds, and is adequate for its purpose. So far as the patients and the wards are concerned the hospital is well up to date, but the kitchen and scullery require reconstruction, and there is at present no larder worthy of the name. It is remarkable that when the wards were reconstructed and the accommodation was increased by one-third, no thought appears to have been expended upon the requirements for adequately providing for the needs of an increased number of patients. It was quite remarkable to find, after visiting so attractive a hospital from the patients' point of view, that the kitchen, and especially the larder, should be utterly unworthy of the institution in its present improved and enlarged condition. It is quite evident that the writer, who wrote in defence of St. Albans as a cathedral city with an up-to-date hospital, could not have first inspected the kitchen and what is called the larder. We feel confident that another year will not be allowed to pass before the hospital committee takes the domestic offices in hand, and by the sweeping away of the obstruction now caused by the coal-cellars, secures the reconstruction of the larder and the kitchen, and certain necessary hygienic improvements in the lavatories for the servants, and the children's wards.

We were most courteously received by the matron, Miss Macdonald, who was evidently keenly interested in every part of the hospital. She has laboured successfully to bring it up to its present state of efficiency. We understand that the mortuary is, in fact, the *post-mortem* room also, and that there is no chapel or view-room in connection with it. Every well-administered hospital has its view-room or chapel, and this is a point which we commend to the earnest attention of the St. Albans and Mid-Herts Hospital authorities.

### HARROW COTTAGE HOSPITAL.

This institution was established in 1872, and for thirty-five years it did most excellent work in the old hospital, which was one of the earlier specimens of cottage hospitals. In its organisation and regulations it was admittedly one of the best. In 1907 the old hospital was vacated and a new hospital was built. A really excellent site, with a very large garden, was purchased, and it was determined to erect the hospital on a declivity just above the garden. We inspected this new hospital with pleasurable anticipation on October 27, 1913. It was evidently designed by someone obsessed by the Gothic architectural idea. Indeed, an inspection of the buildings shows that that idea has been ridden to death, for the institution which has resulted, save only in the case of the wards and their adjuncts, is not up to date as a hospital, and no care appears to have been taken to introduce the most ordinary labour-saving features. In the upper storey, for example, everything has been sacrificed to give the building exteriorly a picturesque roof so designed as to interfere with the practical usefulness and convenience of practically every room, and the approach to every room, occupied by the staff or used for the administrative purposes of the institution. The actual waste of money thus caused must be considerable. In other directions the comforts of the inmates, the facilities for service and for the handling of the patients seem to have been disregarded or ignored. For instance, the staircases are so narrow as barely to admit of two persons walking abreast, and they are so constructed as to render it practically impossible, without risk of accident, to carry a patient up or down, should occasion arise. The passage-way to the apartments occupied by the resident staff is rendered ridiculous and inconvenient by the roof. The food-lift is so planned and placed as to cause continuous difficulties in loading and unloading it. If the architect is wholly responsible he should be called to book and be invited to bear the cost of making the necessary reconstructions that the buildings may serve adequately the smooth working of a modern hospital. It is a great deal too bad that a hard-working and excellent committee, such as this hospital has always had the good fortune to possess, should have had their work and that of their staff so hampered by the design and planning of parts of the new hospital. We have no knowledge who is responsible for the present plan, but the present buildings should be brought up to the standard of a modern hospital at once.

The wards and those portions of the hospital in connection with the ward units are, in many cases, excellent, though they lack cross-ventilation and are unnecessarily high. The kitchens are unusually large and have ample means for up-to-date cooking at a minimum cost. The beds, linen, and general fittings, as far as they exist, are good, and the same may be said of the operation theatre. We noticed, however, that the sister in charge, who has been there for eleven years, has no comfortable sitting-room to herself, yet the present condition of the hospital testifies to her excellent efficiency. It is remarkable that Miss E. Peake's name is not given in the report, although two pages are taken up with the names of other workers for the hospital. We hope, in justice, this omission will be remedied in the next annual report.

The annual income, we are glad to notice, is more than sufficient to meet the expenditure. It is a good feature, too, that the patients' payments amounted to upwards of £130 for the last year, 1912. The district nurses, though still under the management of the same committee as that of the Cottage Hospital, no longer have their quarters at the hospital, and are kept wholly distinct from the hospital organisation. This change, we understand, is due to the fact that they are Queen's nurses, and that the rules of that organisation provide that they shall be under no authority but that of their own organisation.

The rules of the Harrow Cottage Hospital during the period that it occupied the old buildings were taken as a model by many cottage hospitals, and deservedly so. These fundamental rules and by-laws were, however, amended in February 1911, and we must leave the examination of these rules for description in a separate article. We found everything in excellent order throughout the hospital at the time of our visit, October 27, 1913, and were much struck by the alertness and manifest ability exhibited by the members of the nursing staff.

**The Royal Surrey County Hospital, Guildford, will be reported on next week.**

